# Electronic health literacy and related factors among nursing students: a latent profile analysis

**DOI:** 10.3389/fpubh.2026.1845664

**Published:** 2026-05-20

**Authors:** Liangmei Xu, Na Li, Xia Ding, Haiyan Wang, Pin Yin, WenTing Pan

**Affiliations:** 1 Anhui No.2 Provincial People’s Hospital, Hefei, China; 2 School of Nursing, Anhui Institute of Medicine, Anhui, China

**Keywords:** eHealth literacy, health literacy, influencing factors, latent profile analysis, nursing students

## Abstract

**Background:**

In the context of rapid information technology development and widespread internet-based healthcare, electronic health literacy (eHL) is essential for health promotion and disease prevention. As prospective nurses, nursing students require sufficient eHL to retrieve, evaluate, and apply digital health information in clinical practice and health education. However, evidence regarding the heterogeneity and influencing factors of eHL among nursing interns in China remains limited.

**Methods:**

This multicenter cross-sectional study recruited 1,191 nursing interns from six hospitals in Anhui Province using convenience cluster sampling. Data were collected from October to December 2025. Latent profile analysis, multivariate logistic regression, and Spearman’s rank correlation were applied.

**Results:**

Three distinct latent classes of eHL were identified: low eHL (28.89%), medium eHL (45.42%), and high eHL (25.69%). The overall mean eHL score among the interns was 77.57 (SD = 14.68). Multivariate logistic regression analysis indicated that, compared with those in the low eHL group, nursing students with higher educational levels, higher health information literacy, and fewer weekly episodes of staying up late were more likely to be classified into the medium and high eHL groups (*p* < 0.05). Furthermore, Spearman’s rank correlation analysis revealed that eHL was positively correlated with health information literacy (*p <* 0.05).

**Conclusion:**

Electronic health literacy among nursing students remains at a moderate level. Universities should strengthen systematic health literacy education, guide students to access reputable health websites, and recommend reliable health information resources. Hospitals may establish health consultation centers or virtual platforms to assist nursing students in identifying and evaluating online health information and addressing their practical health needs.

## 1 Introduction

According to the “Healthy China 2030” Planning Outline issued by the Central Committee of the Communist Party of China and the State Council, the construction of a “Healthy China” has been elevated to a national strategic priority, recognizing population health as the cornerstone of national prosperity, socioeconomic development, and public well-being (1). The central government has explicitly stated that “improving the health literacy of all people is one of the most fundamental, economical, and effective measures to enhance the health of the population” (2). Information is regarded as a critical conduit to health (3). As a central dimension of health literacy, health information literacy (HIL) comprises a spectrum of competencies, including identifying health information needs, locating reliable information sources, retrieving and evaluating information quality, and applying information to inform health-related decisions (4). Distinct from general health literacy, HIL integrates both health literacy and information literacy, emphasizing the discovery and utilization of health-related information (5). Elevated HIL not only enables individuals to acquire knowledge regarding disease prevention and treatment, maintain healthy lifestyles, and enhance doctor–patient communication, but also reduces healthcare resource waste and promotes broader economic and societal progress (6). As the backbone and reserve workforce of society, university students exert a profound and far-reaching influence on public health behaviors; therefore, enhancing their HIL is of paramount importance for both individual well-being and population health.

Nevertheless, health information acquisition and decision-making in contemporary society are increasingly mediated by digital environments, making it insufficient to consider HIL independently of the digital context in which it is enacted. In the Web 2.0 era, the rapid digitalization of daily life has made eHealth a critical driver of health promotion, with eHealth literacy (eHL) emerging as an indispensable extension of health literacy in the digital domain (7). According to data from the China Internet Network Information Center (CNNIC), as of June 2025, the number of internet users in China reached 1.123 billion, with an internet penetration rate of 79.7% (8). As more individuals turn to digital resources to support health-related decision-making, the challenge of information quality has grown more acute, as online health information is characterized by vast quantities, rapid proliferation, diverse sources, and highly variable quality (9). In this complex digital environment, university students must navigate unverified and often conflicting health information online, necessitating a construct that captures not only general information competencies but also the ability to critically evaluate and apply information within digital contexts. eHealth literacy addresses this need. It is defined as the ability to find, understand, and critically evaluate health-related information from electronic resources and to apply that knowledge to address health problems (10). Unlike HIL, which emphasizes informational competencies broadly, eHL explicitly integrates digital navigation skills with critical appraisal and practical application in online environments, making it the most suitable construct for examining health information behaviors among university students in digital settings.

College students represent one of the most digitally engaged demographics, exhibiting a clear preference for sourcing health information via online platforms (11). Recently, the integration of artificial intelligence (AI) has become increasingly prevalent in navigating healthcare systems, interpreting medical data, and seeking personalized health advice. While AI facilitates AI-augmented lifelong learning, translation, simplification, and content filtering (12), it simultaneously escalates the complexity of the digital information landscape. This evolution requires individuals to possess a higher level of eHL to distinguish the accuracy of algorithmic suggestions and to reduce the risks associated with complex error messages and information overload. Despite these technological advancements, current evidence indicates that the overall eHL level of Chinese college students still needs improvement, and significant differences in digital ability exist across disciplines (13). Medical students, benefiting from formal medical curricula and early exposure to evidence-based practice, generally demonstrate higher eHL scores than their peers in non-medical fields (14). Nevertheless, even medical students frequently exhibit deficiencies in critically appraising online resources or translating digital information into informed health decision-making (15). As future healthcare providers, nursing students must master these core competencies to effectively conduct patient education and optimize health outcomes.

Norman and Skinner (16) developed the inaugural eHealth Literacy Scale (eHEALS), which has since been translated into over a dozen languages (including Chinese, Japanese, Korean, and Dutch) and extensively validated across diverse populations. Furthermore, researchers have developed localized instruments tailored to specific cohorts. For instance, Tang et al. (17) established the eHealth Literacy Scale for Chinese College Students by integrating the Lily model with the original eHEALS framework, resulting in a 20-item tool encompassing three dimensions: eHealth acquisition, appraisal, and practice. However, a unified standard for classifying eHL levels among college students remains to be established. In a survey of 626 nursing students (freshmen and sophomores) on campus, Shi (18) reported an overall mean eHL score of 69.41 (SD = 3.48), characterizing the participants’ eHL as moderate. Although these students could retrieve health information online, they often struggled to discern authentic content from misinformation. Notably, as medical education progresses and clinical knowledge accumulates, medical students tend to become more proficient in acquiring and evaluating high-quality eHealth information and applying it to health maintenance (19–21). Based on this progression, we believe that as course learning and clinical practice progress, the eHL skills of nursing interns will further improve.

**Figure 1:**
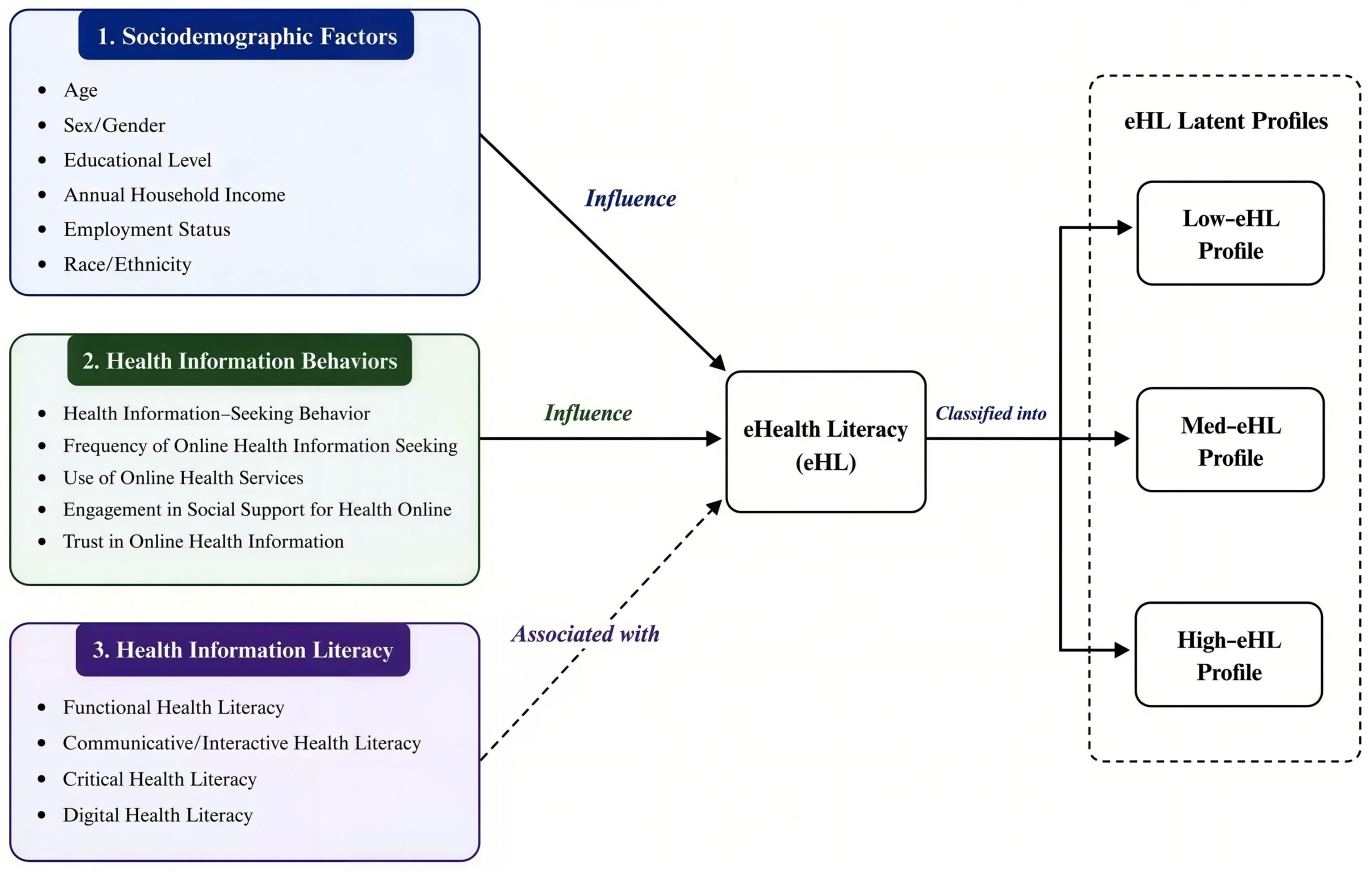
Hypothesized conceptual model.

In summary, further research is warranted to identify nursing students with low eHL and to explore associated factors, including sociodemographic characteristics and HIL. Latent profile analysis (LPA) is a robust method for classifying participants into distinct subgroups based on specific variables (22). Therefore, this study aimed to identify distinct subgroups of eHL among nursing interns and to analyze the factors associated with these subgroups. The hypothesized conceptual model is presented in Figure 1. The research questions were as follows:

1. What is the level of eHL among nursing students participating in clinical practice?
2. What are the characteristics of students with relatively low eHL?
3. How do sociodemographic characteristics and HIL influence the different subgroups of nursing interns?

## 2 Methods

### 2.1 Study design

This was a multicenter cross-sectional study.

### 2.2 Study setting and participants

From October to December 2025, a cross-sectional survey was conducted among nursing interns from six tertiary Grade A hospitals in Anhui Province, China, using a convenience cluster sampling approach. To enhance regional and institutional representativeness, two hospitals were selected from southern, central, and northern Anhui, respectively, including both general hospitals and university-affiliated hospitals. Within each selected hospital, all nursing interns on duty during the study period were invited to participate. The inclusion criteria were: (1) nursing students currently engaged in clinical internships; (2) full-time undergraduate or higher vocational nursing students; and (3) provision of informed consent and voluntary participation. The exclusion criteria were: (1) absence from clinical practice for 1 month or longer due to sick leave or personal leave; and (2) prior clinical internship experience (e.g., students enrolled in bridging programs from associate to bachelor level).

### 2.3 Sample size

The sample size was estimated based on Kendall’s recommendation (23), which suggests that the number of observations should be at least 10 times the number of variables. In this study, a total of 59 variables were included (17 demographic variables and 42 variables related to HIL and eHL). Considering an anticipated invalid questionnaire rate of 20%, the minimum required sample size was calculated as 738 using the formula: 59 × 10/0.8. For latent class analysis (LCA), it is generally recommended that each latent class include at least 50 participants to ensure reliable classification (24). Based on previous studies indicating that eHL is typically categorized into no more than three latent classes, a minimum sample size of 150 was required for this analysis. Ultimately, a total of 1,191 valid responses were obtained, exceeding the minimum requirements for both general statistical analyses and LPA, thereby ensuring adequate statistical power and model stability.

### 2.4 Data collection instruments

#### 2.4.1 Demographic characteristics

A self-designed questionnaire was used to collect baseline data from study participants, including demographic characteristics (e.g., gender, ethnicity, place of origin, educational background), academic and family circumstances, physical and mental health status, and sources and frequency of health information acquisition.

#### 2.4.2 Health information literacy

The Health Information Literacy Questionnaire for Medical Students was developed by Jin et al. (5). It comprises five dimensions: health information acquisition, cognition, evaluation, application, and ethics. Each item is rated on a 5-point Likert scale ranging from “strongly disagree” to “strongly agree,” with scores assigned from 1 to 5. Higher total scores indicate higher levels of HIL. The questionnaire demonstrated excellent internal consistency, with a Cronbach’s *α* coefficient of 0.955.

#### 2.4.3 Electronic health literacy

The eHealth Literacy Scale for College Students (19) was used to assess eHL among college students. The scale comprises three dimensions: eHealth information acquisition ability (7 items), evaluation ability (8 items), and application ability (5 items), with a total of 20 items. All items are rated on a 5-point Likert scale ranging from “strongly disagree” to “strongly agree,” with scores from 1 to 5. Higher total scores indicate higher levels of eHL. The scale demonstrated good internal consistency, with a Cronbach’s *α* coefficient of 0.915.

### 2.5 Data collection

Prior to data collection, the investigators contacted internship coordinators at each participating hospital to explain the study objectives, significance, and survey procedures. The electronic questionnaire was distributed to nursing intern group leaders via the WeChat platform, who then forwarded it to eligible participants. Standardized instructions were provided to ensure consistency, outlining the survey content and completion requirements. Participants were required to provide informed consent by selecting an agreement option before proceeding, after which the survey timer was activated. All items in the questionnaire were set as mandatory, and each IP address was restricted to a single submission to minimize duplicate responses. A total of 1,254 questionnaires were distributed. Based on pilot testing, the minimum completion time was estimated to be 3 min; therefore, 24 questionnaires with completion times of less than 3 min were excluded. In addition, 39 questionnaires exhibiting patterned responses were identified and removed through a combination of manual review and Excel-based logical checks. Ultimately, 1,191 valid questionnaires were retained, yielding an effective response rate of 94.98%.

### 2.6 Data analysis

LPA was conducted using Mplus version 8.3, while SPSS version 26.0 was used for group comparisons and regression analyses. Continuous variables were described as mean and standard deviation if normally distributed, or as median and interquartile range [M (P25, P75)] if not normally distributed. Categorical variables were expressed as frequencies and percentages (%). Group differences were examined using the chi-square test or nonparametric tests (Kruskal–Wallis test), as appropriate. In the LPA, the 20 items of the eHealth Literacy Scale were included as observed indicator variables and treated as continuous variables based on their Likert-scale measurement properties. Model fit for LPA was evaluated using multiple indices. Lower values of the Akaike Information Criterion (AIC), Bayesian Information Criterion (BIC), and adjusted BIC (aBIC) indicate better model fit. Entropy ranges from 0 to 1, with values closer to 1 reflecting higher classification accuracy. The Lo–Mendell–Rubin likelihood ratio test (LMRT) and the bootstrap likelihood ratio test (BLRT) were used to compare models with *k* and *k*-1 profiles; a significant *p*-value (*p* < 0.05) indicates that the model with k profiles provides a significantly better fit. Class probabilities represent the proportion of individuals assigned to each latent class within the sample, and classes with proportions below 5% were considered potentially unstable (25). In addition, Spearman’s rank correlation analysis was performed to assess the association between eHL and HIL.

### 2.7 Ethical considerations

Ethical approval for this study was granted by the Ethics Committee of Anhui No.2 Provincial People’s Hospital (Approval (R) 2025–117) on October 16, 2025. The study objectives were communicated to all participants. Informed consent was obtained from all participants, who were informed that their data would be handled confidentially and anonymously.

## 3 Results

### 3.1 Participant characteristics

A total of 1,191 nursing students were included in this study, with an average age of 20.35 (SD = 0.84) years. Among them, 1,092 (91.5%) obtained health information less than 5 times per week, 75 (6.2%) obtained it 5 to 10 times per week, and 24 (2.3%) obtained it more than 10 times per week. When feeling unwell, 500 (41.98%) would first consult the internet, 157 (13.18%) would turn to parents or friends, and 534 (44.84%) would consult a doctor. Other details are presented in Table 1.

**Table 1:** Participants’ demographic characteristics (*N* = 1,191).

Categorical Variables	*N*	%
Gender		
Male	177	14.86
Female	1,014	85.14
Ethnicity		
Han	1,167	97.98
Minority	24	2.02
Place of Origin		
Rural	951	79.84
Urban	240	20.16
Only child		
Yes	206	17.30
No	985	82.70
Class leader		
Yes	335	28.13
No	856	71.87
Chronic illness		
Yes	109	9.15
No	1,082	90.85
Smoking		
Yes	30	2.52
No	1,161	97.48
Drinking		
Yes	66	5.54
No	1,125	94.46
Academic qualification		
Junior and below	749	62.89
Bachelor or above	442	37.11
Family per capita monthly income		
<1,000¥	1,150	96.55
1,000–5,000¥	32	2.68
>5,000¥	9	0.77
Self-assessed health status		
Poor	37	3.11
General	663	55.67
Good	491	41.22
Study pressure		
Small	40	3.36
General	808	67.84
Large	343	28.80
Physical exercise/week		
Never	288	24.18
1–3 times	790	66.33
>3 times	113	9.49
Staying up late/week		
Never	200	16.79
1–3 times	722	60.62
>3 times	269	22.59

### 3.2 Latent profile analysis of eHL among nursing students

The LPA was conducted using the scores of the 20 eHEALS items as manifest variables. Models with one to five latent classes were sequentially estimated (Table 2). As the number of classes increased, the values of AIC, BIC, and aBIC progressively decreased, indicating improved model fit. The three-class model demonstrated the highest entropy value (0.980), suggesting excellent classification accuracy. In addition, both the LMRT and BLRT were statistically significant (*p* < 0.05), indicating that the three-class model provided a significantly better fit than the two-class model. Although the four- and five-class models showed further reductions in AIC, BIC, and aBIC values, the five-class model yielded a non-significant LMRT result (*p* = 0.293), suggesting no significant improvement over the four-class model. Moreover, the five-class solution contained a very small class (0.8%), indicating potential instability. The four-class model, while statistically acceptable, resulted in less distinct class separation and reduced interpretability. In contrast, the three-class model showed adequate class proportions (28.9, 25.7, and 45.4%), high posterior probabilities (all >98%), and clear interpretability. Therefore, considering model fit indices, classification accuracy, class size, and parsimony, the three-class model was selected as the optimal solution.

**Table 2:** Model fitting indexes for eHL in nursing students.

Class	AIC	BIC	aBIC	Entropy	LMRT(P)	BLRT(P)	Probabilities of classes
1	59882.689	60085.991	59958.936	1.000	–	–	–
2	46151.656	46461.692	46267.933	0.976	<0.001	<0.001	0.395/0.605
3	37615.203	38031.972	37771.509	0.980	0.001	<0.001	0.289/0.257/0.454
4	36052.719	36576.222	36249.055	0.971	0.001	<0.001	0.220/0.150/0.379/0.251
5	34570.972	35201.208	34807.337	0.975	0.2933	<0.001	0.008/0.229/0.139/0.373/0.251

AIC, Akaike’s Information Criterion; BIC, Bayesian Information Criterion; LMR-LR, Lo–Mendell–Rubin adjusted likelihood Ratio Test; BLRT, Bootstrap Likelihood Ratio Test.

### 3.3 Characteristics and classification of latent profiles

**Figure 2:**
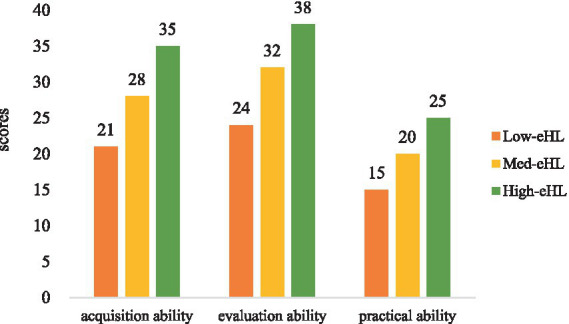
Score characteristics of each item in different latent profiles.

Based on the scores of each latent profile across the eHEALS items, the categories were designated as follows (Figure 2): Profile 1 (*n* = 344, 28.89%) had the lowest median scores on all dimensions (e.g., acquisition: median = 21, IQR 21–23; evaluation: median = 24, IQR 24–25; application: median = 15, IQR 15–16), all below the 25th percentile of the total sample. Therefore, this group was classified as the “low eHL” group. Profile 2 (*n* = 541, 45.42%) showed moderate median scores (acquisition: median = 28, IQR 27–28; evaluation: median = 32, IQR 30–32; application: median = 20, IQR 19–20), falling between the 25th and 75th percentiles, and was labeled the “medium eHL” group. Profile 3 (*n* = 306, 25.69%) exhibited the highest median scores (acquisition: median = 35, IQR 28–35; evaluation: median = 38, IQR 32–40; application: median = 25, IQR 20–25), above the 75th percentile, and was defined as the “high eHL” group.

### 3.4 Univariate analysis of factors influencing latent profiles of eHL among nursing students

Using latent profile classifications as the dependent variable, univariate analysis revealed statistically significant differences across profiles in factors including education, class leader, only child, self-assessed health status, physical exercise, weekly frequency of staying up late, weekly health information search frequency, and preferred help-seeking channel when ill (*p* < 0.05), as shown in Table 3. Significant variations were also observed in HIL and domain-specific scores among different latent profiles of eHL (*p* < 0.05), as presented in Table 4.

**Table 3:** Univariate analysis of different latent profiles.

Categorical variable	Group			*χ*^2^/*H*	*P*
	Low eHL (*n* = 344)	Medium eHL (*n* = 541)	High eHL (*n* = 306)		
Gender				3.890^a^	0.143
Male	46	75	56		
Female	298	466	250		
Place of origin				3.591^a^	0.166
Urban	77	96	67		
Rural	267	445	239		
Academic qualification				190.483^b^	<0.001
Junior and below	251	406	92		
Bachelor or above	93	135	214		
Family per capita monthly income				5.929^b^	0.052
<1,000¥	339	519	292		
1,000–5,000¥	4	19	9		
>5,000¥	1	3	5		
Only child				6.089^a^	0.048
Yes	54	85	67		
No	290	456	239		
Class leader				9.98^a^	0.007
Yes	93	135	107		
No	251	406	199		
Chronic illness				0.924^a^	0.630
Yes	35	45	29		
No	309	496	277		
Self-assessed health status				54.073^b^	<0.001
Poor	18	11	8		
General	224	318	121		
Good	102	212	177		
Study pressure				7.983^a^	0.092
Small	7	17	16		
General	226	379	203		
Large	111	145	87		
Physical exercise/week				62.779^b^	<0.001
Never	127	129	32		
1–3 times	192	370	228		
>3 times	25	42	46		
Staying up late/week				147.171^b^	<0.001
Never	8	92	100		
1–3 times	206	336	180		
>3 times	130	113	26		
Smoking				4.408^a^	0.110
Yes	12	15	3		
No	332	526	303		
Drinking				0.383^a^	0.826
Yes	19	32	15		
No	325	509	291		
Health information search frequency/week				35.523^b^	<0.001
<5 times	322	514	256		
5–10 times	16	22	37		
>10 times	6	5	13		
Preferred help-seeking channel when ill				36.741^a^	<0.001
Doctor	121	237	176		
Network	167	225	108		
Parents or friends	56	79	22		

^a^Chi-square test, ^b^Kruskal–Wallis test.

**Table 4:** Comparison of the HIL scale for nursing students in different latent profiles.

Continuous variable	*n* = 1,191	Low-eHL (*n* = 344)	Med-eHL (*n* = 541)	High-eHL (*n* = 306)	*H*	*P*	*Post hoc* comparisons
eHL	80(67, 90)	60(60, 64)	80(76, 80)	97(80, 100)	1038.726	<0.001	C1 < C2 < C3
eHL acquisition	28(24, 33)	21(21, 23)	28(27, 28)	35(28, 35)	968.277	<0.001	C1 < C2 < C3
eHL evaluation	32(25, 36)	24(24, 25)	32(30, 32)	38(32, 40)	1010.068	<0.001	C1 < C2 < C3
eHL application	20(16, 22)	15(15, 16)	20(19, 20)	25(20, 25)	946.827	<0.001	C1 < C2 < C3
HIL	88(79, 100)	70(66, 81)	88(85, 92)	102(92, 110)	767.412	<0.001	C1 < C2 < C3
HIL acquisition	19(16, 21)	15(15, 17)	20(17, 20)	21(20, 25)	598.738	<0.001	C1 < C2 < C3
HIL cognition	16(14, 19)	12(12, 15)	16(16, 17)	19(17, 20)	639.35	<0.001	C1 < C2 < C3
HIL evaluation	20(17, 23)	15(15, 18)	20(19, 21)	24(21, 25)	711.941	<0.001	C1 < C2 < C3
HIL application	16(14, 18)	12(12, 15)	16(15, 17)	18(17, 20)	678.09	<0.001	C1 < C2 < C3
HIL ethics	17(16, 20)	14(12, 17)	16(16, 19)	20(19, 20)	509.922	<0.001	C1 < C2 < C3

### 3.5 Multivariate logistic regression analysis of latent profiles

Using latent profiles of eHL (with low eHL as the reference group) as the dependent variable, significant variables from univariate analysis were included as independent variables in a multivariate logistic regression. The results showed that, compared with the low eHL group, nursing students with higher education levels, higher HIL scores, and fewer weekly episodes of staying up late were more likely to belong to the medium and high eHL groups. Details are presented in Table 5.

**Table 5:** Multivariate logistic regression analysis of different latent profiles.

Profile	Variable	*B*	SE	Wald	*P*	OR	95%CI
C2	Constant	−13.692	1.286	113.396	<0.001		
	HIL	0.163	0.011	208.770	<0.001	1.178	1.152 ~ 1.204
	Junior and below	−0.229	0.221	1.079	0.299	0.795	0.516 ~ 1.226
	Only child	0.303	0.274	1.226	0.268	1.354	0.792 ~ 2.317
	Class leader	0.079	0.217	0.132	0.716	1.082	0.707 ~ 1.656
	Self-assessed health status (good as the control group)						
	Poor	−0.376	0.542	0.481	0.488	0.687	0.238 ~ 1.986
	General	−0.062	0.206	0.092	0.762	0.940	0.628 ~ 1.406
	Physical exercise/week (>3 times as the control group)						
	Never	−0.254	0.394	0.415	0.519	0.776	0.359 ~ 1.678
	1–3 times	0.001	0.368	0.000	0.997	1.001	0.486 ~ 2.062
	Staying up late/week (>3 times as the control group)						
	Never	1.575	0.450	12.258	<0.001	4.833	2.001 ~ 11.673
	1–3 times	0.316	0.208	2.303	0.129	1.371	0.912 ~ 2.062
	Health information search frequency/week (>10 times as the control group)						
	<5 times	0.927	0.834	1.238	0.266	2.528	0.493 ~ 12.953
	5–10 times	0.582	0.954	0.372	0.542	1.789	0.276 ~ 11.604
	Preferred help-seeking channel when ill (Parents or friends as the control group)						
	Doctor	0.131	0.280	0.219	0.640	1.140	0.658 ~ 1.975
	Network	−0.351	0.274	1.645	0.200	0.704	0.411 ~ 1.204
C3	Constant	−33.851	2.128	253.144	<0.001		
	HIL	0.383	0.020	376.262	<0.001	1.467	1.411 ~ 1.525
	Junior and below	−1.642	0.315	27.153	<0.001	0.194	0.104 ~ 0.359
	Only child	0.531	0.395	1.807	0.179	1.700	0.784 ~ 3.686
	Class leader	0.308	0.331	0.865	0.352	1.360	0.711 ~ 2.600
	Self-assessed health status (with “good” as the reference group)						
	Poor	0.599	0.845	0.502	0.479	1.820	0.347 ~ 9.532
	General	−0.232	0.309	0.565	0.452	0.793	0.433 ~ 1.452
	Physical exercise/week (>3 times as the control group)						
	Never	−0.438	0.603	0.528	0.468	0.645	0.198 ~ 2.105
	1–3 times	−0.096	0.521	0.034	0.853	0.908	0.327 ~ 2.519
	Staying up late/week (>3 times as the control group)						
	Never	2.092	0.579	13.038	<0.001	8.101	2.602 ~ 25.216
	1–3 times	0.767	0.388	3.915	0.048	2.154	1.007 ~ 4.607
	Health information search frequency/week (>10 times as the control group)						
	<5 times	−0.343	1.088	0.100	0.752	0.709	0.084 ~ 5.984
	5–10 times	0.172	1.219	0.020	0.888	1.188	0.109 ~ 12.953
	Preferred help-seeking channel when ill (Parents or friends as the control group)						
	Doctor	−0.058	0.493	0.014	0.907	0.944	0.359 ~ 2.481
	Network	−0.444	0.489	0.823	0.364	0.642	0.246 ~ 1.674

### 3.6 Correlation between eHealth literacy and health information literacy

Spearman’s rank correlation analysis indicated that eHL was significantly and positively correlated with HIL and all its dimensions (*p* < 0.05; Tables 6, 7). The correlation coefficients between eHL and the dimensions of HIL ranged from moderate to strong (*r* = 0.666–0.835, *p* < 0.05). In addition, significant positive correlations were observed among all dimensions of HIL (*r* = 0.594–0.933, *p* < 0.05).

**Table 6:** Correlations among eHL dimensions.

Continuous variables	eHL	Acquisition	Evaluation	Application
eHL	1.000			
Acquisition	0.937**	1.000		
Evaluation	0.958**	0.853**	1.000	
Application	0.933**	0.829**	0.884**	1.000

**p < 0.01.

**Table 7:** Correlations between eHL and HIL.

Continuous variables	eHL	HIL	Acquisition	Cognition	Evaluation	Application	Ethics
eHL	1.000						
HIL	0.835**	1.000					
Acquisition	0.745**	0.879**	1.000				
Cognition	0.761**	0.921**	0.800**	1.000			
Evaluation	0.798**	0.933**	0.773**	0.852**	1.000		
Application	0.784**	0.895**	0.724**	0.773**	0.820**	1.000	
Ethics	0.666**	0.826**	0.594**	0.725**	0.751**	0.748**	1.000

**P < 0.01.

## 4 Discussion

In clinical nursing practice, the ability to obtain high-quality health information from the internet, accurately assess its reliability, and guide patients’ health decisions based on it has become an indispensable core skill (23). As future healthcare providers and role models for healthy lifestyles, improving eHL among nursing students is significant for their learning of relevant professional knowledge, enhancing their personal physical and mental health, disseminating health knowledge, and improving public health status.

Assessment scales are the primary tools used in eHL research. In this study, the eHealth Literacy Scale for College Students developed by Tang et al. (17) at Fudan University was utilized. This scale is specifically tailored for the Chinese undergraduate population and was validated in a sample of 1,163 college students in Shanghai. It demonstrated strong psychometric properties, with a Cronbach’s *α* of 0.915 and factor loadings ranging from 0.451 to 0.829; consequently, it has been widely adopted in domestic eHL research. However, the scale lacks predefined cut-off scores for proficiency levels and was originally developed in the context of Web 1.0. It may not fully capture the interactive and participatory aspects of health information processing characteristic of the Web 2.0/3.0 era. Although Wu et al. (26) developed a mobile-based eHL scale (m-eHEALS) adapted for Web 2.0 technologies, its validity requires further empirical verification. To advance the quality of eHL research in China, future studies should focus on refining measurement dimensions and enhancing the context-specific applicability of these instruments.

The results of this study showed that the total eHEALS score for nursing students in Anhui Province was 77.57 (SD = 14.68), representing a moderate level of eHL. This average is comparable to the score of 79.8 (SD = 10.5) reported for medical students (27). However, it is significantly higher than the mean score of 69.77 (SD = 10.45) reported for non-medical college students (28). These comparisons suggest that while nursing students have a stronger foundation in eHL than their non-medical peers, there remains significant room for improvement to reach high proficiency. This discrepancy may be associated with the medical curriculum, as medical education tends to foster greater health consciousness among students. Furthermore, as professional courses deepen and medical knowledge accumulates, nursing students may become more adept at identifying high-quality electronic health information and applying it to health maintenance (29, 30). This suggests that in future curriculum design, it may be beneficial to integrate content such as online health knowledge and channels for obtaining online health information to promote the improvement of eHL among college students.

Among the three dimensions of eHL, the practical ability dimension had the lowest average score, indicating that nursing students struggle with practical skills in this area. On the one hand, they lack the ability to retrieve and obtain health information from professional databases. On the other hand, they also lack the ability to apply health information to solve real-life problems. Yuan and Wu (31) also pointed out that the majority of Chinese college students are unable to conduct advanced information retrieval and lack the ability to apply health information effectively. Catherine et al. (32) believes that college students should increase their use of journal literature databases rather than relying solely on internet search engines to find health information. Niu and Li (33) found that medical students also face deficiencies in applying electronic health information and have low levels of trust in online information, which affects their use of electronic health information. Therefore, in the new media environment, nursing students should recognize the positive impact of eHL in promoting their own health and actively engage in eHL education to enhance their knowledge, search skills, and application abilities, ultimately improving their own eHL.

This study found that the eHL of nursing students in Anhui Province can be categorized into three latent profiles: low eHL (28.89%), medium eHL (45.42%), and high eHL (25.69%). The educational qualifications of nursing students in the low eHL group were mostly at the junior college level or below. This may be related to differences in educational methods and vocational education compared to regular universities in China (34). The short duration of junior college education, high course density, and focus on basic theories and skill training, with less emphasis on eHL education, could explain these differences. Li et al. (35) found that undergraduates have significantly higher willingness and ability to obtain and utilize online information than junior college students. The main difference is that undergraduate education integrates digital information resources into the curriculum system more systematically and continuously. Therefore, it is suggested that junior colleges should focus on eHL education, incorporate health-related topics in public courses such as “Literature Retrieval,” and embed eHL skills into pre-class activities, case discussions, and assignments in core courses (36).

Chinese college students not only exhibit unhealthy lifestyle habits but also show characteristics typical of the college stage (37). For example, frequent late-night activities, excessive internet use, and sedentary behavior (38). Li et al. (39) conducted a survey of 305 college students and found that only 8.2% of them stayed up late. This study found that nursing students who do not stay up late are more likely to belong to the high eHL group. Relevant studies suggest that groups with higher eHL are more likely to adopt healthy behaviors, such as maintaining regular physical activity, adhering to healthy living habits, and undergoing regular check-ups. Conversely, groups with lower eHL are more likely to develop poor health habits (40, 41). Government strategies aimed at improving the health outcomes of young people often promote the use of peer-led interventions. Peer-led health promotion is defined as “the teaching or sharing of health information, values, and behaviors by members of similar age or status groups” (42). The perceived advantages of this approach stem from the high level of interaction among peers, which facilitates the sharing of health information in relatable ways (43). Schools can strengthen dormitory management, promote healthy living habits, and discourage late-night studying or prolonged use of electronic devices. Students with high health literacy can be trained as “health promotion volunteers” to spread healthy behavioral habits in smaller groups, such as dormitories and classes, particularly for issues like staying up late and prolonged sitting.

The HIL is at the core of health literacy. The results of this study show that individuals with higher eHL tend to have better abilities in accessing, understanding, evaluating, and applying health information. This may be related to the fact that nursing students with higher HIL are more willing or capable of utilizing online information resources to search for the health information they need and are better at distinguishing and screening relevant information to meet their own needs. The online environment is complex, and the quality of health information varies significantly (44). Wang (45) found that health information on online platforms is often highly commercialized, poorly readable, and presented in a single format. College students lack the ability to understand complex professional knowledge, which further complicates the acquisition and application of health information. Authoritative information sources are vital for obtaining reliable health information. In recent years, the development of formal medical platforms, such as health applications, online medical platforms, and hospital websites, has provided valuable channels for the public’s online health information searches (46). Relevant departments should introduce policies and regulations, increase supervision of the online environment, purify cyberspace, and intervene promptly to address low-quality information. Online platforms should diversify the presentation of health information and make it easier to understand. Furthermore, expanding the channels for providing health information and encouraging more professionals to contribute to online information provision is essential.

## 5 Limitations

This study has several limitations that warrant consideration. First, as a cross-sectional design was employed, it limits the ability to draw causal inferences between the variables examined. Second, as in prior research, reliance on self-reports to assess nursing students’ eHL may affect the accuracy of the results. Future studies are encouraged to develop and utilize objective tools to more accurately evaluate nursing students’ eHL. Third, the sample had a much higher proportion of female (85.14%) than male (14.86%) nursing interns, potentially limiting the generalizability of the findings to male populations. Although the proportion of male nurses in China increased from 2.4 to 3.4% between 2016 and 2020, indicating a continued but slow upward trend, the gender gap remains substantial (47). Of note, gender was not significantly associated with eHL group membership, which is consistent with prior findings (48). Nevertheless, given the very small proportion of male participants in this study, caution is warranted when interpreting this null finding, and future research with a more balanced gender composition is needed to confirm the absence of gender differences in eHL. Fourth, all questions in the questionnaire were mandatory. While mandatory questions can increase response rates, they may also lead to less genuine or incomplete answers, especially on sensitive topics. Moreover, forced responses may cause discomfort or resistance among participants, potentially affecting the quality of the answers. Future studies could consider incorporating optional questions to improve data accuracy and participant engagement. Fifth, the research subjects in this study were limited to Anhui Province, and the use of convenience sampling at the hospital level may limit the generalizability of the findings. Future studies could expand the scope of the investigation, include a larger sample size, and improve both the representativeness of the sample and the generalizability of the conclusions. Despite these limitations, the study employed Latent Profile Analysis (LPA) to identify the latent categories of eHL among nursing students, providing a theoretical foundation for developing targeted strategies and interventions to enhance their eHL.

## 6 Conclusion

The findings of this study indicate that the overall eHL level among nursing students in Anhui Province is moderate, with significant potential for improvement. The eHL of nursing students is heterogeneous and can be categorized into three distinct latent profiles: “low eHL group,” “medium eHL group,” and “high eHL group.” Higher educational attainment, better HIL, and fewer episodes of staying up late per week were associated with higher probabilities of belonging to the medium- and high-level eHL profiles. Moreover, a significant positive correlation was observed between eHL and HIL. These results emphasize the importance of developing targeted educational strategies to enhance eHL, particularly by improving students’ abilities to critically evaluate and practically apply digital health information. Tailored interventions should be designed for different student subgroups, with particular attention to those in the low eHL category. Additionally, promoting healthy lifestyle habits and incorporating eHL training into the nursing curriculum are recommended to better prepare future nurses for the increasingly digital healthcare environment.

## Data Availability

The raw data supporting the conclusions of this article will be made available by the authors, without undue reservation.
