# Correction: Electronic health literacy and related factors among nursing students: a latent profile analysis

**DOI:** 10.3389/fpubh.2026.1886442

**Published:** 2026-06-02

**Authors:** Liangmei Xu, Na Li, Xia Ding, Haiyan Wang, Pin Yin, WenTing Pan

**Affiliations:** 1 Anhui No.2 Provincial People's Hospital, Hefei, China; 2 School of Nursing, Anhui Institute of Medicine, Anhui, China

**Keywords:** eHealth literacy, health literacy, influencing factors, latent profile analysis, nursing students

In the published article, there was an error in the affiliation order for all authors.

It previously stated:

“^1^Blood Purification Center, Anhui No.2 Provincial People's Hospital, Hefei, China

^2^School of Nursing, Anhui Institute of Medicine, Anhui, China

^3^Department of Nursing, Anhui No.2 Provincial People's Hospital, Hefei, China

^4^Department of Respiratory, Anhui No.2 Provincial People's Hospital, Hefei, China”

The correct version is:

“^1^Anhui No.2 Provincial People's Hospital, Hefei, China

^2^School of Nursing, Anhui Institute of Medicine, Anhui, China”

The original article has been updated.

